# Enhanced production and organic solvent stability of a protease from*Brevibacillus laterosporus* strain PAP04

**DOI:** 10.1590/1414-431X20165178

**Published:** 2016-03-18

**Authors:** P. Anbu

**Affiliations:** Department of Biological Engineering, Inha University, Incheon, South Korea

**Keywords:** Brevibacillus, Organic solvent-stable protease, Peptide synthesis, Solvent stability

## Abstract

A bacterial strain (PAP04) isolated from cattle farm soil was shown to produce an extracellular, solvent-stable protease. Sequence analysis using 16S rRNA showed that this strain was highly homologous (99%) to *Brevibacillus laterosporus*. Growth conditions that optimize protease production in this strain were determined as maltose (carbon source), skim milk (nitrogen source), pH 7.0, 40°C temperature, and 48 h incubation. Overall, conditions were optimized to yield a 5.91-fold higher production of protease compared to standard conditions. Furthermore, the stability of the enzyme in organic solvents was assessed by incubation for 2 weeks in solutions containing 50% concentration of various organic solvents. The enzyme retained activity in all tested solvents except ethanol; however, the protease activity was stimulated in benzene (74%) followed by acetone (63%) and chloroform (54.8%). In addition, the plate assay and zymography results also confirmed the stability of the PAP04 protease in various organic solvents. The organic solvent stability of this protease at high (50%) concentrations of solvents makes it an alternative catalyst for peptide synthesis in non-aqueous media.

## Introduction

Organic solvents are extremely toxic to microorganisms. These chemicals have been shown to cause lysis following cell penetration, owing to disruption of the cell membrane and internal structures (1). However, some bacteria are able to develop stability in organic solvents by various adaptations, such as solvent efflux pumps, rapid membrane repair, lower cell membrane permeability, increased membrane rigidity, and decreased cell surface hydrophobicity (1). Organic solvent tolerance is a strain-specific property, and the toxicity of a solvent correlates with the logarithm of its partition coefficient in *n*-octanol and water (log*P*_ow_) (2); organic solvents with low log *P_ow_* values (1.5-4.0) are considered more toxic than those with higher log*P*_ow_ values (3).

Most bacterial enzymes are both less active and less stable in the presence of organic solvents. Because of enzyme denaturation, peptide synthesis rates are also very low in organic solvents (4). Several methods have been employed to improve enzyme activity and stability in organic solvents, such as chemical modification, immobilization, protein engineering, and directed evolution (5). However, some naturally occurring solvent-tolerant strains are able to produce enzymes that retain their stability and activity in organic solvents without any need for modification or engineering (6). These natural solvent-stable enzymes can be found in bacterial strains collected from the environment (e.g., soil samples), following screening methods to isolate potent strains capable of producing protease in organic solvents.

Proteases catalyze the hydrolysis of peptide substrates in normal aqueous conditions, and the synthesis of peptides in non-aqueous conditions (5,7). For effective industrial applications, enzymes must be active and stable at high concentrations of organic solvents, such that sufficient amounts of product can be recovered while contaminants and side reactions are eliminated (5). Microbial proteases represent one of the largest classes of industrial enzymes, accounting for approximately 40% of the total worldwide sales of enzymes (8). These proteases can be produced in large quantities and genetically manipulated to increase activity much more easily than proteases derived from plants and/or animals (9).

Most organic solvent-stable proteases have been isolated and characterized from gram-negative bacteria (10 11 12 13); however, a few are available from gram-positive bacteria (14,15). The production of solvent-stable proteases by microorganisms can be influenced by factors such as growth media, incubation period, pH, temperature, and sources of carbon and nitrogen (4). Even small improvements in biotechnological enzyme production processes have resulted in greater commercial success. This study describes the isolation of the *Brevibacillus laterosporus* strain PAP04 and the production of an organic solvent-stable enzyme from this strain that, to the best of our knowledge, has not been previously reported.

## Material and Methods

### Isolation of organic solvent-stable microorganisms

Soil samples were collected from cattle farm sites in South Korea. Organic solvent-stable bacteria were isolated from soil samples according to established methods (10). Briefly, 1 g of soil sample was suspended in 10 mL of sterile water by shaking, and 5 mL of this suspension were added to 250 mL bottles containing 25 mL of Lysogeny broth (Sigma, USA) supplemented with toluene and benzene (2.5% v/v each). Culture vessels were sealed with chloroprene rubber stoppers to prevent evaporation of organic solvents and then incubated at 37°C for 72 h on a shaker at 180 rpm. Next, 5-mL aliquots of culture were transferred into fresh media and cultured again under the same conditions. These cultures were diluted and plated onto skim milk agar media (1 g/L yeast extract, 20 g/L agar, 1% skim milk) lacking organic solvents, and then incubated at 37°C for 36 h to screen for protease-producing strains. These strains were purified and screened again on skim milk agar plates for further confirmation.

### Selection of a highly potent solvent-stable strain

Bacteria were inoculated into 25 mL of Lysogeny broth medium and incubated at 30°C for 4 h with shaking at 180 rpm. About 0.5 mL of this culture was transferred into 50 mL of protease production medium (10 g/L peptone, 0.5 g/L (NH_4_)_2_SO_4,_ 0.3 g/L MgSO_4_·7H_2_O, 1 g/L CaCl_2_·2H_2_O, 1 g/L NaCl, 10 mL glycerol, pH 7.0). The inoculated flasks were incubated at 37°C for 48 h with shaking at 180 rpm. After incubation, the culture was centrifuged at 11,100 *g* for 10 min at 4°C.

To obtain a strain capable of highly producing organic solvent-stable proteases, strains isolated from plate screening were further screened with organic solvents (benzene and toluene). Solvents were added to 1 mL of supernatant, to reach a final concentration of approximately 25%, and tubes were covered with aluminum foil. These mixtures were incubated at 37°C for 24 h with shaking at 100 rpm. The residual protease activity was measured as described below. Based on the initial screening by plate assay and subsequent stability tests in organic solvents, the strain PAP04 was selected for further studies.

### Identification of the selected strain by 16S rRNA sequencing

The selected strain PAP04 was identified by 16S rRNA sequencing as follows. Genomic DNA was extracted using a genomic DNA purification kit (Promega, USA) and then used as a template to amplify 16S rRNA sequences by polymerase chain reaction (PCR) using the universal 16S rRNA gene primers: 8-27F, 5'-AGAGTTTGATCCTGGCTCAG-3' and 1472R, 5'-TACGGYTACCTTGTTACGACTT-3'. PCR products were then sequenced, and 16S rRNA gene sequences were compared to other nucleotide sequences by Basic Local Alignment Search Tool (http://www.ncbi.nlm.nih.gov/blast).

### Optimization of solvent-stable protease production

Protease production was assessed at 24-h intervals for incubation times up to 72 h. Cell-free supernatants were collected every 24 h following centrifugation at 11,100 *g* for 10 min at 4°C; these supernatants were used to determine protease activity. The following carbon sources were used at a 1% concentration in growth media: glucose, glycerol, lactose, maltose, and sucrose. Carbon sources were sterilized separately and added aseptically to autoclaved media. The following nitrogen sources were also tested: casein, corn steep liquor, gelatin, peptone, and skim milk. Protease activity was determined at pH values ranging from 6.0 to 11.0 (adjusted prior to autoclaving) and at temperatures ranging from 20 to 60°C.

### Protease assay

Protease activity was measured using a previously described method (16) with modifications. Briefly, a 500-μL aliquot of culture supernatant was mixed with 500 μL of 100 mM Tris-HCl buffer, pH 8.0, containing 1% (w/v) casein (used as a substrate) and incubated for 30 min at 37°C. Reactions were stopped by the addition of 500 μL of 20% trichloroacetic acid and incubated at room temperature for 15 min, followed by centrifugation at 13,300 *g* for 15 min to remove precipitates. The absorbance at 280 nm for each supernatant was determined. One unit of protease activity was defined as the amount of enzyme required to liberate 1 μg of tyrosine in 1 min.

### Effect of organic solvents on the stability of crude protease

Crude protease from supernatants was filtered through a 0.22-μm membrane. Enzyme solutions were placed in screw-capped tubes and mixed with the following organic solvents at a 50% final concentration: acetone, benzene, chloroform, dimethylformamide, dimethyl sulfoxide (DMSO), ethanol, hexane, methanol, isopropanol, and toluene. These mixtures were incubated at 30°C for 2 weeks with shaking at 100 rpm. After incubation, each sample was carefully withdrawn from the solution or aqueous phase, in case of water-immiscible solvents. Residual protease activity was determined as described above; controls contained the enzyme solution lacking organic solvents. Enzyme stability is reported as the protease activity relative to the control.

### Substrate gel electrophoresis analysis

For gelatin zymogram analysis, sodium dodecyl sulfate polyacrylamide gel electrophoresis (SDS-PAGE) was performed according to a previously established method (17), with minor modifications. Samples were electrophoresed on 10% polyacrylamide gels containing 0.1% gelatin. Following electrophoresis, gels were rinsed with 0.25% Triton X-100 and incubated for 1 h at 37°C in 50 mM Tris-HCl buffer, pH 8.0. Protease activity was visualized by staining gels with Coomassie brilliant blue R-250 (Sigma, USA).

## Results

### Isolation and identification of solvent-stable strains

Soil samples were mixed with media containing toluene and benzene (2.5% each), then incubated for 3 days at 37°C and plated on skim milk agar media. A total of 22 bacteria were able to produce clear zones that indicate hydrolysis of substrate, with strain PAP04 showing the highest clear zone. Screening media was supplemented with substrate for selection of potent protease-producing strains. To reconfirm each strain’s stability in solvent, bacteria were inoculated into protease production media and crude enzyme was mixed with toluene and benzene, followed by evaluation of protease activity. Enzyme activity from the PAP04 strain was significantly stable in both solvents; therefore, this strain was selected for further studies.

**Figure 1:**
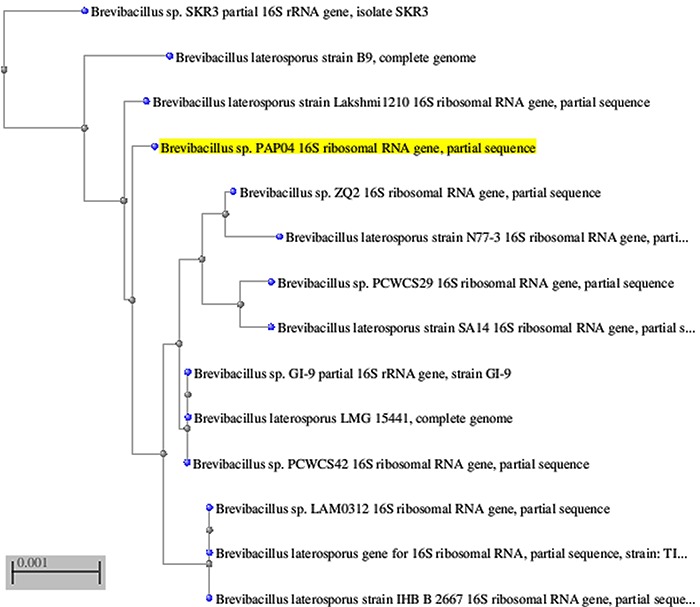
Phylogenetic tree based on *16S* rRNA sequencing, which was generated using the neighbor-joining method. Relationships of the isolated PAP04 sequence (highlighted in yellow) to other*Brevibacillus* species are shown. Bar, 0.001 changes per nucleotide position.

PCR was utilized to amplify the 16S rRNA sequence from the PAP04 strain, which was then purified and used for sequencing. The 16S rRNA sequence (1443 bp) from the strain PAP04 was compared to that from other bacterial species and shown to exhibit a high similarity (99%) with *B. laterosporus* LMG15441 by phylogenetic tree analysis (Figure 1).

### Organic solvent-stable protease production

**Figure 2:**
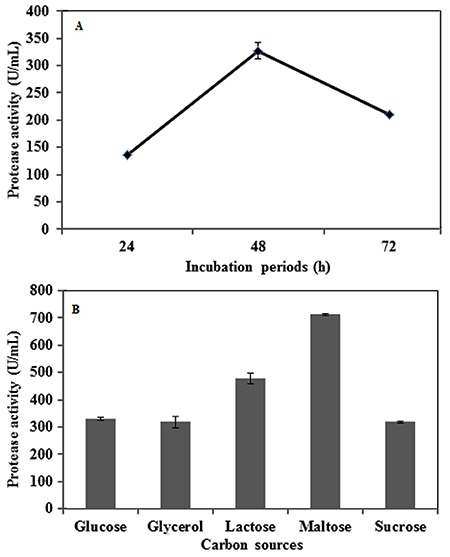
*A*, Effect of incubation periods on organic solvent-stable protease production. *B*, Effect of various carbon sources on organic solvent-stable protease production. Data are reported as means±SE of three independent experiments.

**Figure 3:**
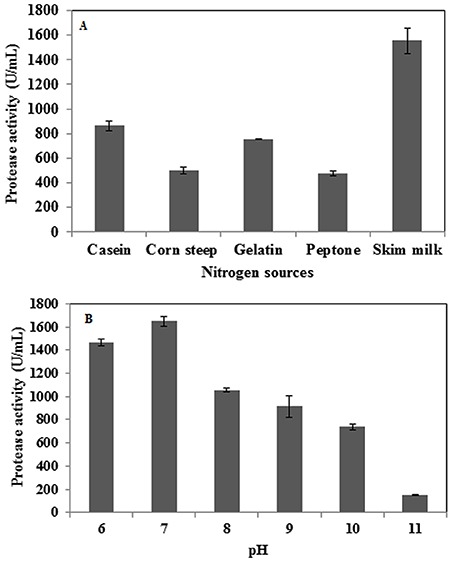
Effect of various nitrogen sources (*A*) and pH (*B*) on organic solvent-stable protease production. Data are reported as means±SE of three independent experiments.

**Figure 4:**
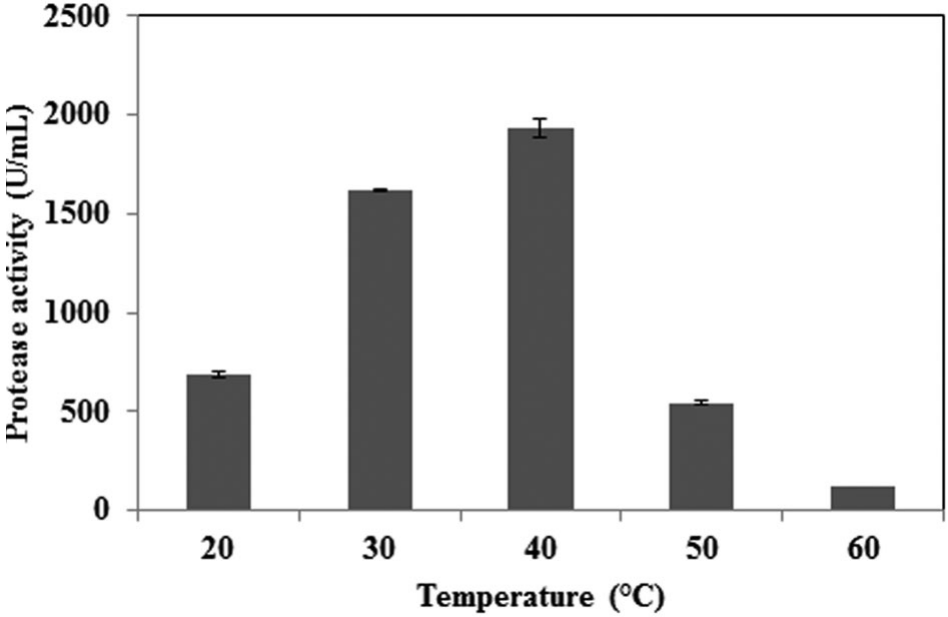
Effect of different temperatures on organic solvent-stable protease production. Data are reported as means±SE of 3 independent experiments.

Culture conditions were optimized to enhance the level of protease production. The selected strain PAP04 was cultured on protease production medium and demonstrated enzyme activity up to 72 h, with maximum protease production occurring at 48 h of incubation (Figure 2A). The effect of various carbon sources on solvent-stable protease production is shown in Figure 2B: glucose, glycerol, and sucrose yielded low levels of protease, while maltose was able to increase protease production significantly; lactose yielded an intermediate effect. The effect of different nitrogen sources was also investigated to optimize protease production in media containing maltose as the carbon source. Skim milk was determined to be the most effective nitrogen source to improve the level of protease production in these conditions (Figure 3A), while other nitrogen sources, such as casein and gelatin, yielded only moderate levels of protease, and corn steep and peptone inhibited protease production. Of the various pH values tested, significant protease production was observed from pH 6 to pH 8, with the highest enzyme activity observed at pH 7.0 (Figure 3B). Protease activity decreased as pH levels increased above 8.0 (Figure 3B); at highly alkaline pH (11), enzyme activity decreased by approximately 90%. Among the various temperatures tested, the highest enzyme activity was observed at 40°C (Figure 4); enzyme activity was significantly reduced when the temperature was increased above this level.

### Stability of crude protease in various organic solvents

**Figure 5:**
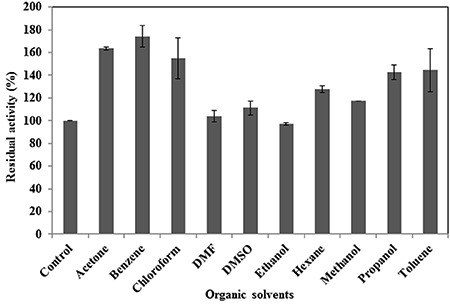
Effect of various organic solvents on the stability of protease. Data are reported as means±SE of 3 independent experiments. DMF: dimethylformamide; DMSO: dimethyl sulfoxide.

**Figure 6:**
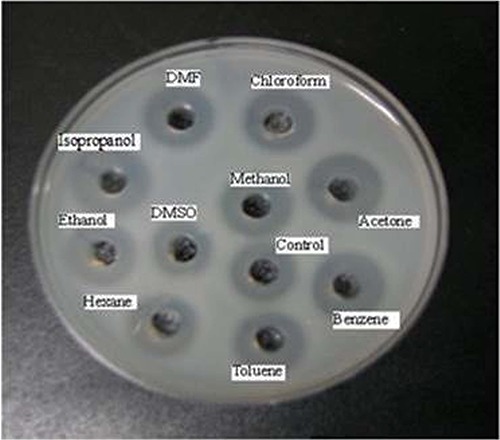
Effect on protease stability in various organic solvents at 50% concentrations, based on a plate assay. DMF: dimethylformamide; DMSO: dimethyl sulfoxide.

**Figure 7:**
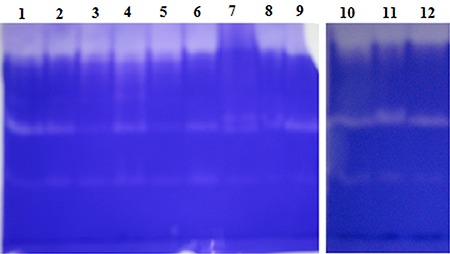
Zymogram analysis of protease activity. *Lane 1*, chloroform (log *P*_ow_ 2.0); *lane 2*, acetone (log*Pow*</emph> -0.24); *lane 3*, benzene (log *Pow* 2.0); *lane 4*, toluene (log *Pow* 2.5); *lane 5*, hexane (log*Pow* 3.6); *lane 6*, ethanol (log*Pow* -0.24); *lane 7*, propanol (log*Pow* 0.05); *lane 8*, dimethylformamide (log *Pow* -1.0); *lanes 9 and 12*, control (without any solvent); *lane 10*, methanol (log *Pow* -0.76); *lane 11*, dimethyl sulfoxide. (log *Pow*-1.35).

Protease stability was assessed in the presence of various organic solvents (at a 50%concentration) with log *P*_ow_ values ranging from -0.24 to 3.6. Residual activity was maintained at a 100% level (compared to that of media lacking solvent) or greater following incubation in all tested organic solvents (Figure 5), except ethanol, which otherwise maintained a significant level of stability (97.1%). Several solvents (acetone, benzene, chloroform, DMSO, hexane, methanol, isopropanol, and toluene) increased enzyme activity, in particular benzene, acetone, and chloroform, which yielded 74, 63, and 54.8% increases in activity, respectively (Figure 5). Enzyme stability was also confirmed by plate assay and zymogram analysis using the same samples. For plate assays, solid medium was prepared with skim milk as the substrate; clear zones indicating hydrolysis of this substrate were observed for all solvent-treated samples to a level similar to or greater than control (Figure 6). Zymography analysis confirmed that the protease stability was maintained in organic solvents; two clear protease bands present in the control lane were also present in all lanes bearing samples treated with organic solvents (Figure 7). These results confirmed that the protease produced by strain PAP04 demonstrated induced activity and enzyme stability in the presence of hydrophobic and hydrophilic solvents.

## Discussion

To isolate solvent-stable bacterial strains, soil samples were cultured in media containing solvents such as toluene and benzene, which were selected for solvent-tolerant bacteria. Protease-producing strains were screened on the basis of the hydrolysis of substrate (skim milk) on agar plates. Among the 22 isolated strains, strain PAP04 showed the largest clear zone around colonies. This potent strain was further selected on the basis of organic solvent stability, as it was found to be significantly stable in the presence of toluene and benzene. Strain PAP04 was identified as *B. laterosporus* by the 16S rRNA sequencing method.

Protease production is generally influenced by nutritional factors (carbon and nitrogen sources) and environmental conditions (pH, temperature, and incubation periods). There have been several reports on protease production by various bacteria (18 19 20 21), including those that have investigated the production of organic solvent-stable proteases (4,6). In our study, peak protease production was observed at 48 h of incubation, after which activity likely decreased owing to nutrient depletion. In other studies, bacteria produced a high level of protease from 48 to 72 h of incubation (6,13,15). Culture conditions, particularly carbon and nitrogen sources, play an important role in stimulating the synthesis of organic solvent-stable proteases. The requirement for specific carbon sources differs from strain to strain, and in this study, maltose was found to increase protease production. The presence of maltose as a carbon source in culture media was also shown to enhance protease production by *Pseudomonas aeruginosa* PseA (22). In addition, PAP04 protease activity was increased approximately 2.2-fold in the presence of skim milk, reflecting the positive effects of this nitrogen source that have also been shown in*Pseudoalteromonas arctica* PAMC 21717 (23) and *Bacillus* sp. N.40 (24). These results confirm that the specific nitrogen source is also critical to improve protease production.

Temperature is another important factor in enzyme synthesis. Most studies have reported that organic solvent-stable bacterial growth and protease production is optimal at temperatures less than 30°C, while some studies have utilized high temperatures to increase both the rate of biotransformation reactions and the solubility of otherwise water-immiscible substrates (25). The low level of protease production observed at high temperatures in our study is likely due to the thermoliability of the protease. Our results were similar to Gupta and Khare (4), who reported an optimum pH of 7.0 for protease production and decreased enzyme synthesis with increasing alkalinity. After optimization of media and culturing conditions, the yield protease was increased approximately 5.91-fold compared to standard conditions.

Most bacteria are not stable in the presence of organic solvents, as these chemicals enter into bacteria and destroy the cell membrane, causing cell lysis (26). However, some gram-positive bacteria possess mechanisms to tolerate organic solvents, such as induction of general stress regulation, production of organic solvent-deactivating enzymes, and formation of endospores (27,28). In the present study, the enzyme produced by *B. laterosporus* PAP04 was stable in all tested hydrophilic and hydrophobic solvents, which were assayed at 50% concentrations. Of note, the solvents benzene, chloroform, and acetone actually increased enzyme activity. Organic solvent-stable enzymes are more attractive for many industrial applications, and most proteases are stable only in some hydrophilic or hydrophobic solvents (6,11). The log*P*_ow_ value is defined as the logarithm of its partition coefficient in a standard *n*-octane/water biphasic system (29). Interestingly, benzene and chloroform have the same log*P*_ow_ values, and similar levels of induced protease activity were observed with these two solvents in our assays. In contrast, some research has reported completely different stabilities in these solvents for proteases produced by*Pseudomonas* species (10,13). Feng et al. (30) reported that enzyme activity can be induced at log *P*_ow_ values above 4.0, while approximately 58-65% of activity is lost at log *P*_ow_ values less than 1.0; a similar effect was also observed with an organic solvent-stable protease produced by *P. aeruginosa* (11).

Biocatalysis in non-aqueous media has several advantages, such as high solubility of hydrophobic substrates, reduced microbial contaminants, and reusability (6). Natural organic solvent-stable enzymes are useful for various applications employing organic solvents as reaction media, as they can be used without any modifications to stabilize the enzymes (5). Several studies have reported enzyme stability in the presence of low concentrations of solvents (4,15,30). However, high concentrations of solvents (above 50%) are required to reduce unwanted hydrolysis during synthesis of peptides and esters (31). Plate assays and zymography analysis also confirmed the stability of the PAP04 protease in multiple organic solvents. These results indicate that the protease from *B. laterosporus* PAP04 was highly suitable for various industrial applications, mainly for peptide synthesis in non-aqueous media.
